# Investigating the Role of Citric Acid as a Natural Acid on the Crystallization of Amoxicillin Trihydrate

**DOI:** 10.1021/acsomega.3c04965

**Published:** 2023-09-20

**Authors:** Hulya Celik Onar, Mustafa Fatih Ergin, Hasniye Yasa

**Affiliations:** † Engineering Faculty, Department of Chemistry, Istanbul University-Cerrahpasa, 34320 Avcılar, Istanbul, Türkiye; ‡ Engineering Faculty, Department of Chemical Engineering, Istanbul University-Cerrahpasa, 34320 Avcılar, Istanbul, Türkiye

## Abstract

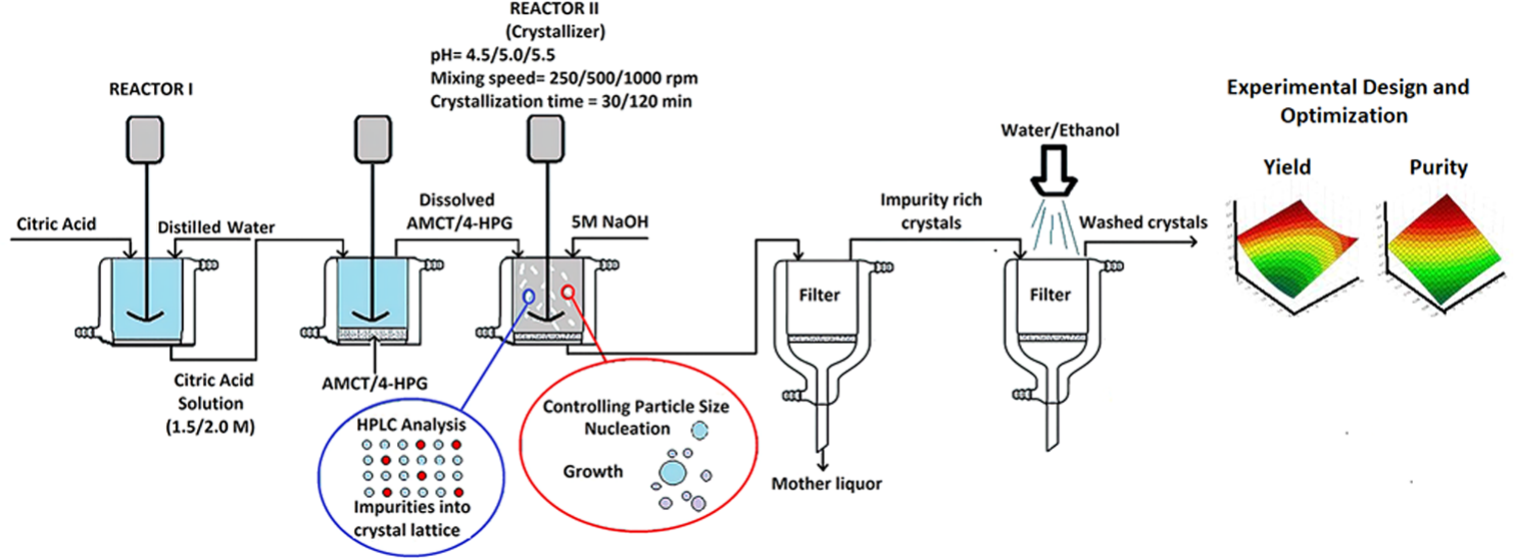

This study investigates the use of environmentally friendly citric acid as the main player in the process, rather than as an additive, to remove impurities from amoxicillin trihydrate (AMCT) crystals, aiming to optimize their purity and yield. By manipulating the concentration of citric acid, mixing speed, crystallization time, and pH, the researchers conducted experiments using a full factorial design. The dissolution stage was analyzed in both batch and continuous crystallization processes, emphasizing the significance of citric acid in enhancing crystallization. HPLC analyses were performed on the resulting crystals, and the data were analyzed using the Multi-Vari Chart program. The findings demonstrated that higher citric acid concentrations positively affected the yield, while factors such as crystallization time, mixing speed, and pH also contributed to the increased yield. The crystals obtained exhibited desirable dimensions sought after in the pharmaceutical industry, eliminating the need for additional purification steps. This study showcased the potential of citric acid in AMCT crystallization, offering advantages in product design, purification, and synthesis. The optimized conditions included a citric acid concentration of 2.0 M, mixing speed of 1000 rpm, crystallization time of 120 min, and pH of 5.5. Notably, the developed process proved to be environmentally friendly by avoiding the use of harmful chemicals, serving as a green alternative for crystallization processes, and producing purer AMCT products. Overall, this research contributes to the existing literature by highlighting the efficacy of citric acid in impurity removal and the optimization of AMCT crystal purity and yield.

## 1 Introduction

Crystallization is a separation and purification technique involving a phase change during which a crystalline product is obtained from a solution. The crystallization technique is frequently employed to produce various products in the food and pharmaceutical industries. Particularly within the pharmaceutical sector, nearly 90% of all active pharmaceutical ingredients (APIs) consist of crystalline organic molecules.^1^ Crystallization holds paramount importance in the pharmaceutical industry, as it serves as a separation process for finished and intermediate products, often marking the final step in active pharmaceutical ingredient (API) production. Downstream processes such as filtration, drying, and grinding, integral to the crystallization process, significantly influence the size, shape, and structure of the obtained crystals, along with the physical and chemical properties of the crystal structure, including the dissolution rate and solubility.^2,3^ The crystal structure emerges through nucleation and crystal growth. During the nucleation stage, molecules within the solution aggregate and form nuclei. Subsequently, in the crystal growth phase, these nuclei develop into macroscopic crystals, attaining a specific size within a defined time frame. The kinetics and mechanism of crystallization are governed by solubility, supersaturation, diffusion, temperature, and the presence of impurities.^4^

The literature predominantly focuses on the adverse impact of introducing impurities structurally similar to the primary molecule on crystal growth. The separation of AMCT from its own degradation products, in particular, has garnered emphasis.^5−9^ In their investigation, Feng et al. demonstrated that an increase in the concentration of degradation products leads to a decrease in AMCT nucleation.^6^ In another study, Ghassempur et al. detailed the integration of impurities into the AMCT crystal lattice and its consequent impact on the process. They established a clear correlation between rising pH levels and declining 4-hydroxyphenylglycine (4-HPG) concentration.^10^ Furthermore, as per separate research, the addition of l-phenylalanine as an impurity at 0.01% (by weight) to l-alanine resulted in a decrease of over half in l-alanine’s growth rate.^11^ The deleterious impact of such impurities on crystal growth can be attributed to their adsorption onto crystal surfaces. Due to the similarity in the molecular structure, impurities can adhere to growth regions, forming structures that hinder the interaction of other crystal molecules.^12^ These differing impurities significantly decelerate the crystal’s growth rate.^13−17^

In their study on anthranilic acid crystallization, Simone et al. noted that the addition of benzoic acid during crystallization affects the crystal structure.^18^ In their research, Kitamura et al. demonstrated the impact of l-phenylalanine on l-glutamic acid growth. They stated that the (110), (111), and (011) surfaces of l-glutamic acid readily adsorb l-phenylalanine through hydrogen bonding.^19^ Similarly, Prasad et al. discussed the presence of phenacetin, which is integrated into the paracetamol crystal lattice. Their study revealed that phenacetin introduced defects in the lattice during crystallization, consequently slowing down the growth rate of paracetamol crystals.^20^ Dowling et al. observed that the additives malonic acid and aspartic acid significantly accelerated the crystal growth rate in their crystallization processes.^21^

Similar to other β-lactam antibiotic drugs, the crystallization of amoxicillin trihydrate (AMCT) also plays a crucial role in controlling the crystalline form, shape, and size. Affordable, widely available, and highly efficient acids such as hydrochloric acid (HCl), sulfuric acid (H_2_SO_4_), and glacial acetic acid (CH_3_COOH) are the most commonly used acids in the purification of AMCT.^5−7,22−24^ However, using these acids raises several environmental, health, and safety concerns due to their extreme corrosiveness and respiratory hazards.^25^ In this study, the utilization of citric acid, a natural organic acid, was explored as an alternative to these acids for the purification of amoxicillin. The goal was to mitigate the harmful effects associated with the use of these acids.

**Figure 1:**
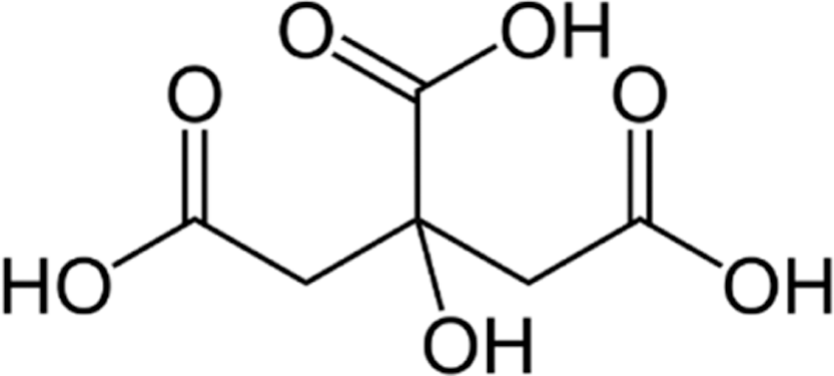
Molecular structure of citric acid.

Citric acid is a weak organic acid, characterized by being odorless, colorless, and a white crystalline powder, with the systematic IUPAC designation 2-hydroxypropane-1,2,3-tricarboxylic acid. It was first discovered by Scheele in 1784 (Figure 1). Due to its nontoxic nature, citric acid finds widespread usage across various sectors. It holds the status of being widely regarded as “GRAS” (generally recognized as safe) and has obtained the seal of approval from the joint FAO/WHO expert committee on food additives.^26^ Furthermore, the acid possesses a food ingredient code, namely, E330 (E331 and E332 for sodium and potassium citrate, respectively), signifying its approval as a food additive for use “in quantum satis” within the European Union.^26^ Citric acid is extensively employed in the food and beverage industries as an acidifier. It enhances the flavors and aromas of fruit juices, ice cream, and marmalades or serves to preserve food due to its antioxidant properties. In the pharmaceutical industry, it finds applications as a blood preservative, an effervescent agent, a pH corrector, an antioxidant to preserve vitamins, and a source of body iron in the form of iron citrate tablets. Additionally, it is used in ointments and cosmetic preparations. In the textile sector, citric acid acts as a foaming agent, aiding in the softening and treatment of fabrics. Within metallurgy, its exceptional chelating ability with various metal ions is harnessed. Metal chelating agents, known for their wide-ranging use in removing heavy metals from soil and plant wastewater, make the most of this property. Owing to its reduced impact on eutrophication, citric acid serves as a substitute for phosphates within the detergent industry.^27−29^

Separation in the chemical and pharmaceutical industries constitutes 40–70% of operating costs. However, separation procedures are extensively employed for the recovery and purification of finished or intermediate products in sectors like pharmaceuticals and food, where the direct impact on human health is significant and substandard products are intolerable. Amoxicillin trihydrate, a semisynthetic antibiotic manufactured in substantial quantities globally, served as the subject of this study.

This study aims to elucidate the role of environmentally friendly citric acid as a solvent in the crystallization process of amoxicillin trihydrate. The effects of citric acid on the nucleation and crystal growth of AMCT were investigated, followed by an analysis of the relationship between efficiency and purity and two different concentrations of citric acid, three distinct mixing speeds, three varying pH values, and two different crystallization times by using a full factorial design. Furthermore, the study delves into particle size analysis and the methodology of crystallizing amoxicillin trihydrate using citric acid.

The data obtained from this study, considered a green process, will contribute not only to the purification of AMCT but also to the development of effective strategies for purifying other β-lactam antibiotics.

## 2 Materials and Methods

### 2.1 Materials

The chemicals used in this study were employed without undergoing any purification process. AMCT was sourced from North China Pharmaceutical Inc., Hebei, China. 4-HPG, ethanol, monopotassium phosphate (KH_2_PO_4_), and dibasic potassium phosphate (K_2_HPO_4_) were procured from Sigma-Aldrich. Citric acid was purchased from Merck, and solutions of 1.5 and 2.0 M citric acid were prepared. Sodium hydroxide was obtained from Sigma-Aldrich, and a 5.0 M NaOH solution was utilized. Distilled water used throughout the study was prepared by using the Milli-Q system.

### 2.2 Methods

#### 2.2.1 Crystallization Process

The 1.5 and 2.0 M citric acid solutions utilized in the crystallization experiments were meticulously prepared using an ultrasonic bath. These solutions were freshly prepared just prior to the experiments and stored in light-shielded containers in a dark environment as they are light-sensitive.

**Figure 2:**
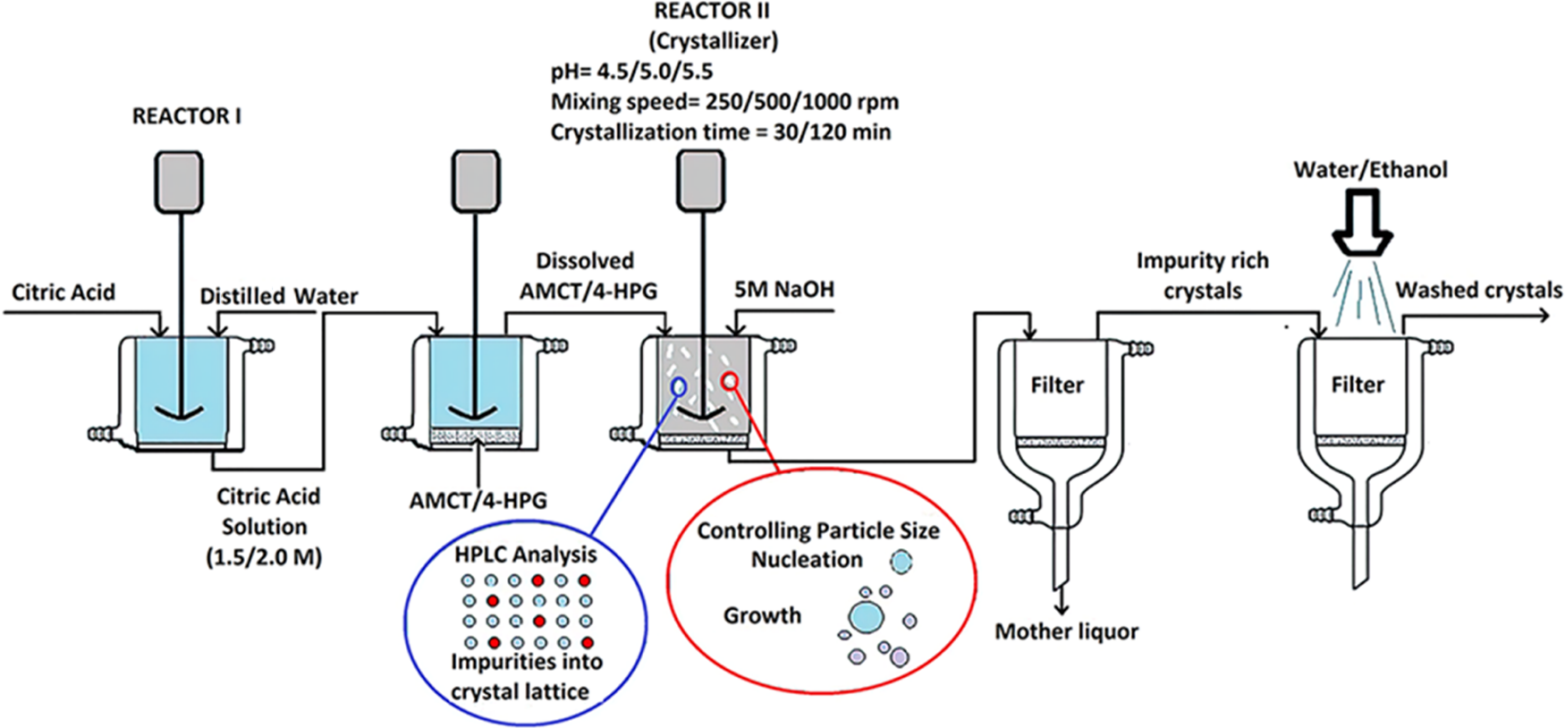
AMCT crystallization process using citric acid.

The AMCT/4-HPG crystallization process using citric acid is illustrated in Figure 2. To standardize the crystallization process, a 100 mL Büchner glass funnel and two 100 mL jacketed reactors were utilized in the experiments. The Büchner glass funnel employed in this study was specially designed to minimize crystal loss, and all experiments were conducted using this apparatus.^5,7^ The process temperature and the temperatures of all solutions used were adjusted to room temperature. Prior to each experiment, the system was operated under the specified conditions for 30 min to attain a stable state. The crystallization process was conducted separately for two different citric acid concentrations (1.5 and 2.0 M), three distinct pH values (4.5, 5.0, and 5.5), three varying mixing speeds (250, 500, and 1000 rpm), and two different crystallization times (30 and 120 min).

For all experiments, 1.67 g of AMCT and 0.167 g of 4-HPG were accurately weighed and placed in the jacketed reactor. The citric acid solution (1.5 or 2.0 M) at room temperature was added to the AMCT/4-HPG mixture in the reactor, and the mixture was stirred at the designated mixing speed (250, 500, or 1000 rpm) until complete dissolution was observed (observed dissolution pH: 1.8). After complete dissolution, stirring was continued under the same conditions for an additional 5 min. Subsequently, the undissolved solids were removed using a 0.45 μm Whatman nylon filter paper. The dissolved AMCT/4-HPG mixture was then adjusted to the desired pH (4.5, 5.0, or 5.5) by gradually adding a 5.0 M NaOH solution at a rate of 1 mL/min while maintaining the same temperature as the system. Two different crystallization times, 30 and 120 min, were employed for crystal formation. The addition of both citric acid and NaOH was continuously monitored by using a pH meter. The resulting fine and delicate crystals were transferred slowly to a Büchner glass funnel and filtered through a 0.2 μm Whatman nylon filter paper. Vacuum pressure was applied to ensure the complete separation of the crystals from the mother liquor. Subsequently, the crystals were transferred to a desiccator for drying after being rinsed with a mixture of 10 mL of ethanol and distilled water (1:1, v/v).

#### 2.2.2 HPLC Analysis

The AMCT crystals prepared using citric acid were subjected to analysis using a Shimadzu 1100 HPLC instrument equipped with a Shimadzu detector and pump. Calibration curves were established for both AMCT crystals and the 4-HPG impurity incorporated into the crystal lattice. The quantities of pure crystals and impurities in the crystal sample were then determined based on the peaks obtained.

A 5 μm Alltech Econosil C-18 column (250 × 4.6 mm) was employed for this study. To prepare the phosphate buffer solution (0.05 M, pH 5.9) utilized in the HPLC analysis, 10 mL of 0.2 M K_2_HPO_4_ and 90 mL of 0.2 M KH_2_PO_4_ were combined and subsequently diluted to a total volume of 1000 mL with deionized water. The entire solution was stirred for 5 min, after which the buffer solution was filtered through a 0.45 μm filter paper. The mobile phase, comprising methanol/acetonitrile (3:1, v/v), was prepared and subsequently filtered. The mobile phase gas was eliminated by using an ultrasonic bath. The HPLC measurements were conducted under the following conditions: flow rate: 1.0 mL/min, wavelength: 230 nm, injector volume: 10 μL, and total run time: 15 min.

The HPLC measurements for the study were performed using the dual gradient elution method, shown in Table 1.

**Table 1:** Dual Gradient Elution Method

time (min)	0	2.5	4	5	7	15
[B/(A + B)]%	0	10	40	20	10	0

A: methanol and B: phosphate buffer solution.

#### 2.2.3 Particle Size Analysis

In this study, samples coded as P_AMCT_ (pure amoxicillin trihydrate), P_30_ (crystals obtained in the 30 min crystallization process), and P_120_ (crystals obtained in the 120 min crystallization process) were utilized. The samples were individually mixed with deionized water in a mass ratio of 1:50. The particle sizes of the samples were determined using a Brookhaven Zeta Potential and Laser Particle Size Analyzer 90Plus Zeta instrument. Each sample was analyzed three times, and the average of the results obtained in this study was used.

#### 2.2.4 Factorial Design

Unintentional errors in an experimental investigation can lead to wasted time, significant financial losses, and compromised empirical accuracy. To thoroughly analyze the effects of employing citric acid as a natural acid on the crystallization of amoxicillin trihydrate, this study successfully conducted experiments within the framework of a well-designed experimental strategy.

Pilot tests were conducted by using various citric acid concentrations, crystallization times, pH values of the crystallization process, and mixing speeds. These tests aimed to establish the correlation between the raw materials (AMCT and 4-HPG) employed in this study and the specific crystallization conditions. Subsequently, the relationship between these pilot studies and the “used citric acid–crystallization conditions” was examined to develop a design for amoxicillin trihydrate crystals.

A “general full factorial” design was employed to evaluate the impact of citric acid and crystallization process conditions on amoxicillin trihydrate crystals. This experimental design facilitated the assessment of multiple factors at more than two levels. The primary objective of using this approach was to yield clearer and more reliable results with a reduced number of trials. Furthermore, this design method aimed to standardize the levels of the crystallization process parameters. The study sought to attain purer and finer-grained AMCT crystals.

In this study, citric acid was utilized in two different concentrations; the crystallization pH took on three distinct values, the mixing speed varied at three rates, and the crystallization time was set at two different durations. The process parameters and corresponding levels employed are detailed in Table 2. A total of 36 trials were conducted for each factor, spanning all levels, using Minitab 19 software. Following these trials, the optimal process conditions were determined for all crystallization processes, taking into consideration the purity and impurity data of amoxicillin.

**Table 2:** Process Parameters and Levels Used for the Experimental Design

	process parameter	level 1	level 2	level 3
1	citric acid concentration	1.5 M	2.0 M	
2	mixing speed	250 rpm	500 rpm	1000 rpm
3	pH	4.5	5.0	5.5
4	crystallization time	30 min	120 min	

The yield and purity of the final product were evaluated following the crystallization process with citric acid. After each trial, crystal samples were subjected to HPLC analysis to assess the process yields and purities. Using the gathered data, the overall yield and purity of the AMCT crystals were calculated.

## 3 Results and Discussion

### 3.1 Particle Size

Crystal size holds great significance in various fields including chemistry, pharmaceuticals, and food. The pharmaceutical industry, in particular, emphasizes the production of specific polymorphs that manifest the desired drug properties. Factors such as the solvent choice, pH, process conditions, and presence of impurities can exert a substantial influence on crystallization outcomes.

In this study, two important factors that affect the growth of AMCT crystals are the impurity (4-HPG) added to the AMCT molecule and the citric acid, in which the AMCT molecule dissolves.

**Figure 3:**
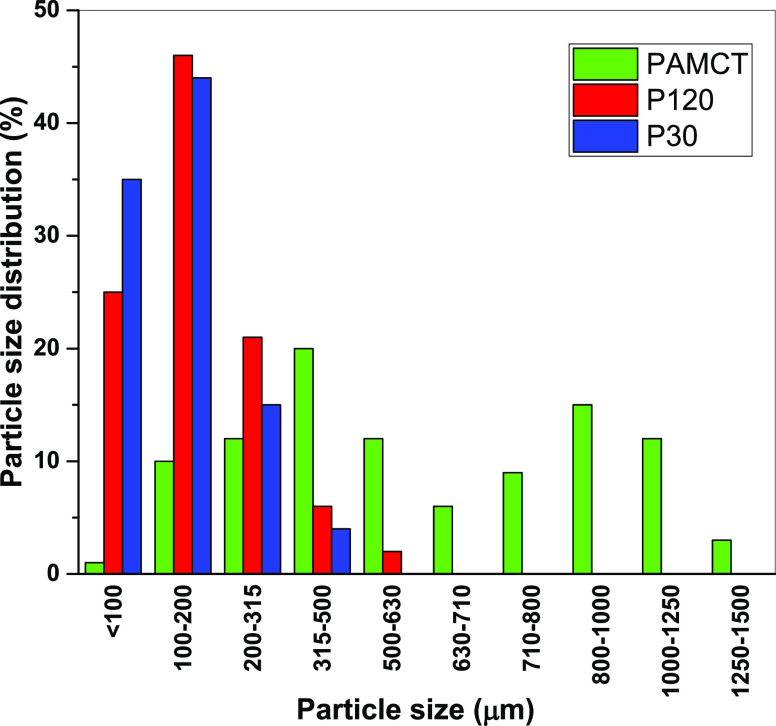
Display of the particle sizes of AMCT samples (P_AMCT_, P_120_, and P_30_).

Particle size analysis was conducted on a total of three samples including P_AMCT_, P_30_, and P_120_. Throughout the entire crystallization process, both P_30_ and P_120_ were visually inspected and examined by using online instruments. As anticipated, an increase in particle size and a broader distribution were observed as the crystallization time of amoxicillin trihydrate extended from 30 to 120 min. Figure 3 displays the particle size distribution comparisons for amoxicillin crystallized with citric acid. It was determined that the average particle diameter of pure amoxicillin trihydrate (P_AMCT_), as measured, ranged between 1250 and 1500 μm, aligning with literature values.^30,31^ For the other samples, the mean particle diameters were found to be 315–500 μm for P_30_ and 500–630 μm for P_120_. In this study, two significant factors influencing the growth of the AMCT crystal are the impurity (4-HPG) introduced to the AMCT molecule and the citric acid in which the AMCT molecule dissolves.

### 3.2 Purity and Yield of AMCT crystals

The calibration curve represents one of the most important methods for quantifying the quantities of active, auxiliary, or impurity substances within the final product. In our study, a calibration curve was employed to determine the quantities of AMCT and impurity substances present in the crystal samples obtained using natural acid. Five standards were utilized for AMCT. The calibration curve was generated using regression analysis to establish the linear relationship between the concentration and peak area. All analyses were conducted in triplicate, and the average values were recorded. The HPLC data acquired were processed to provide comprehensive insight into the efficiency and purity of the crystals obtained through the utilization of natural acid.

#### 3.2.1 Purity of AMCT Crystals

**Figure 4:**
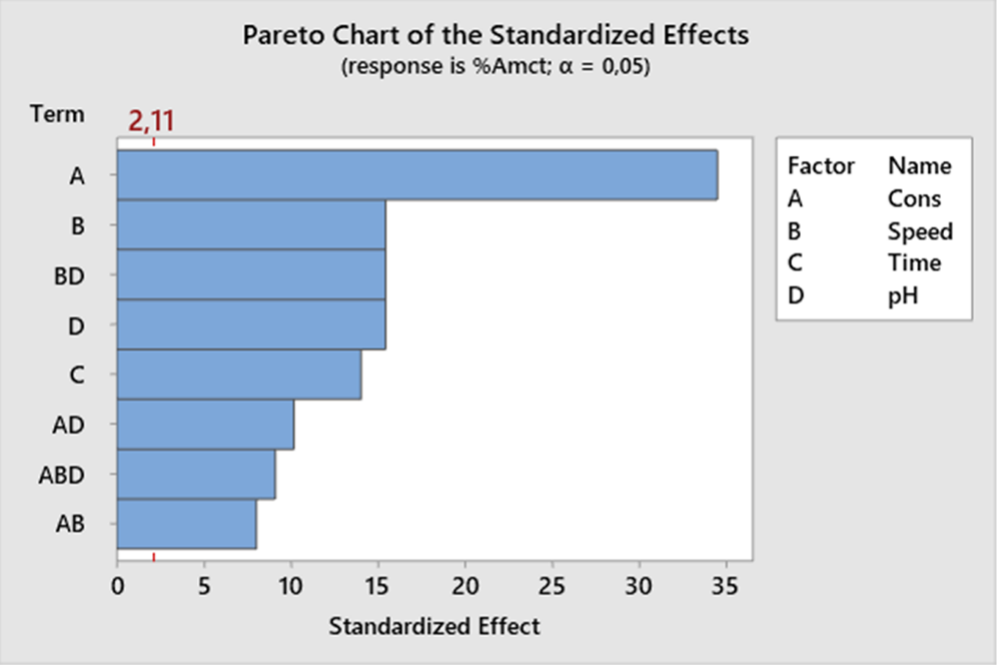
Pareto chart of standardized effects for AMCT purity.

In the crystallization process conducted using natural acid, the purity value of AMCT was modeled according to the full factorial design in Minitab software. The analysis of variance (ANOVA) table for purity values is provided in Table 3. In this table, *P* values below 0.05 were considered significant at a 95% confidence level. As a result, it was determined that the factors Cons, Speed, Time, pH, and the interactions Cons*Speed, Cons*pH, and Speed*pH were significant. Furthermore, by examining the Pareto chart in Figure 4, it can be observed that factors A, B, BD, D, C, AD, ABD, and AD cross the red dotted line, indicating their significance at a 95% confidence level. The summary of the model is presented in Table 4. The standard deviation of residuals (*S*) is 0.00077. The *R*^2^ value indicates that 97.71% of the variation in the response can be explained by the model, which is considered good. The remaining 2.29% of the variation cannot be explained by the model. The adjusted *R*^2^ value (*R*^2^(adj)) is 96.79%, indicating a modified *R*^2^ value. The relatively small difference between *R*^2^ and *R*^2^ (adj) suggests the presence of insignificant factors in the model. The predicted *R*^2^ value (*R*^2^ (pred)) is 99.77%, indicating the predictability of the model for new observations. 1 represents the regression equation in uncoded units.
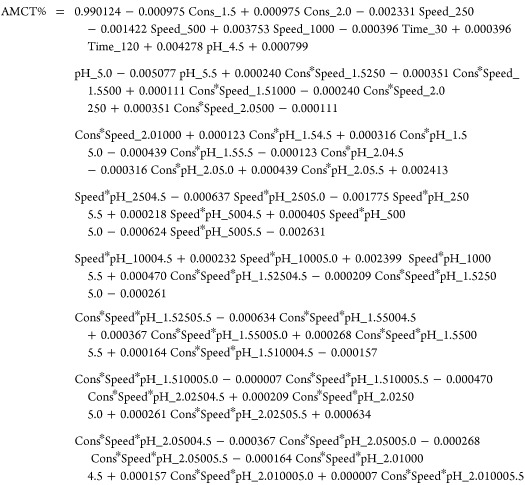
1

**Table 3:** Analysis of Variance for AMCT Purity

source	DF	adj SS	adj MS	*F*-value	*P*-value
model	18	0.000936	0.000052	1803.600	0.000
linear	6	0.000835	0.000139	4827.750	0.000
cons	1	0.000034	0.000034	1186.170	0.000
speed	2	0.000258	0.000129	4483.590	0.000
time	1	0.000006	0.000006	196.3100	0.000
pH	2	0.000537	0.000268	9308.430	0.000
2-way interactions	8	0.000097	0.000012	420.060	0.000
cons*speed	2	0.000002	0.000001	40.180	0.000
cons*pH	2	0.000004	0.000002	64.010	0.000
speed*pH	4	0.000091	0.000023	788.020	0.000
3-way interactions	4	0.000004	0.000001	34.460	0.000
cons*speed*pH	4	0.000004	0.000001	34.460	0.000
error	17	0.000000	0.000000		
total	35	0.000936			

**Table 4:** Model Summary of AMCT Purity

*S*	*R*^2^	*R*^2^ (adj)	*R*^2^ (pred)
0.00017	99.95%	99.89%	99.77%

**Figure 5:**
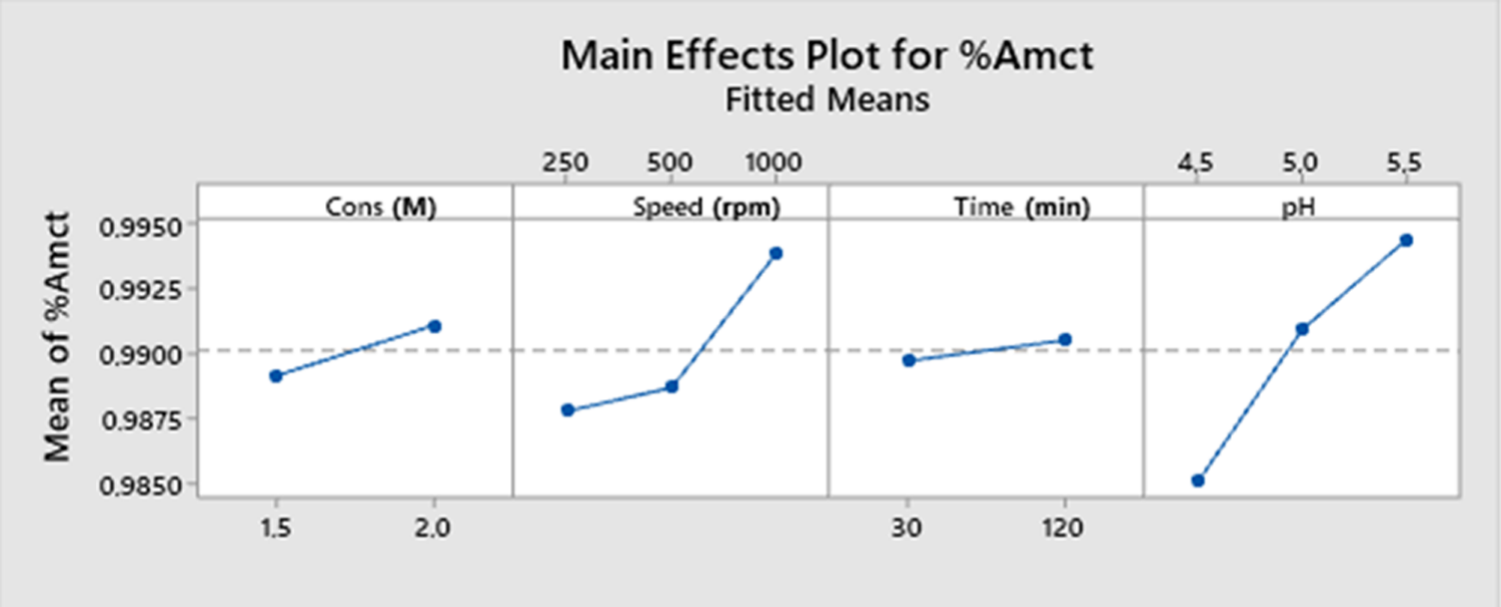
Main effect graph of AMCT purity.

The main effect graph for AMCT purity (Figure 5) examines the independent effects of the natural acid concentration and process variables such as stirring speed, crystallization time, and pH on AMCT purity. The graph clearly shows the relationship between acid concentration and purity. It was determined that as the acid concentration increases, the purity value also increases. This can be explained by the greater removal of 4-HPG, used as an impurity, from the crystal lattice at higher acid concentrations. This statement is supported by the presence of more 4-HPG in the crystal lattice at low acid concentrations, resulting in increased efficiency but decreased purity.

Among the investigated process variables, mixing speed has an effect on AMCT purity, and a range of 250–1000 rpm was studied. When the mixing speed is increased from 250 to 500 rpm, the AMCT purity increases with a small slope. However, when the mixing speed is further increased from 500 to 1000 rpm, the purity increases with a steeper slope.

Another process variable investigated is the crystallization time. Two different times, 30 and 120 min, were applied for the completion of the crystallization process after reaching the predetermined pH value of the solution prepared with natural acid. It was observed that as the expected time for crystallization increases, the purity of the crystal structure also increases.

Another significant variable affecting the crystallization process is the pH. In this study, three different pH values (4.5, 5.0, and 5.5) were preferred. It was found that the pH of the crystallization process affects the amount of 4-HPG incorporated into the AMCT crystal lattice. As the pH of the crystallization process increases, the solubility of 4-HPG also increases. Therefore, it was determined that the amount of 4-HPG in AMCT decreases as the pH of the process increases, resulting in an increase in the AMCT purity.

**Figure 6:**
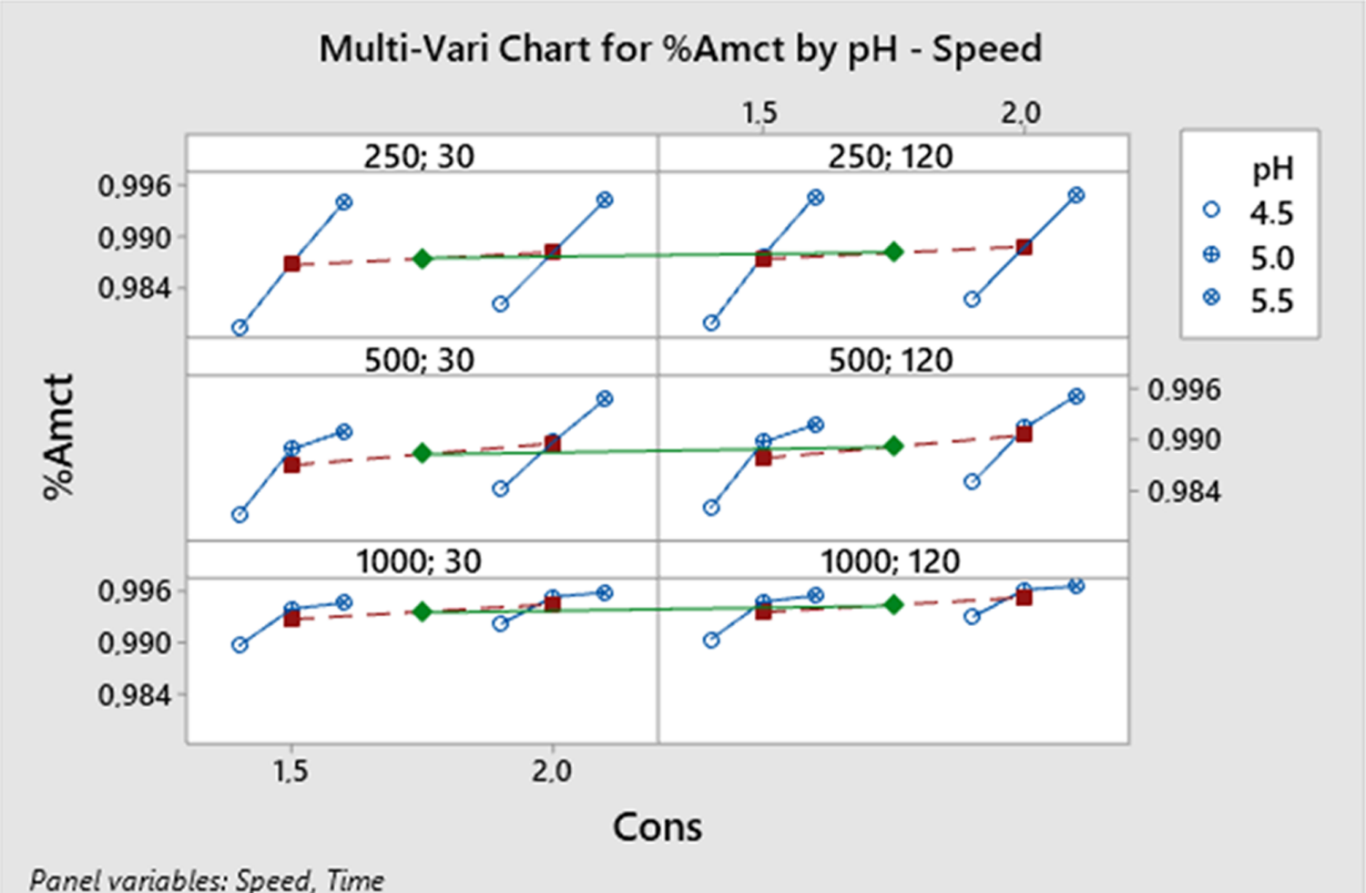
Multieffect plot for AMCT purity.

According to the multiple effect graph shown in Figure 6, the highest purity value of 99.64% was achieved for the combination of pH (5.5), stirring speed (1000 rpm), and crystallization time (120 min).

**Figure 7:**
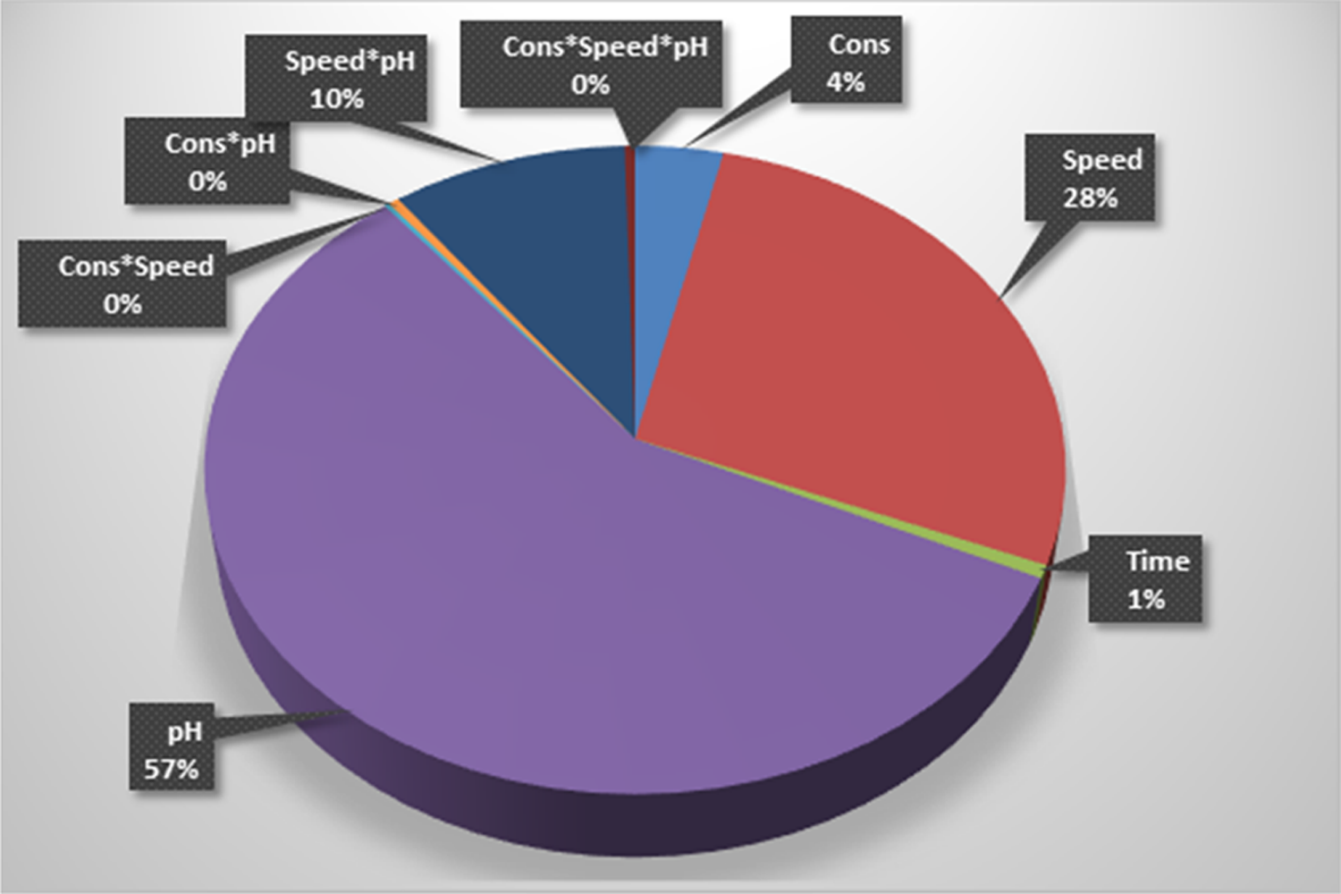
Factors affecting AMCT purity.

The factors influencing purity in the crystallization process are presented in the pie chart in Figure 7. Out of the total variability, pH contributes 57%, mixing speed contributes 28%, natural acid concentration contributes 4%, and crystallization time contributes 1%. Regarding pairwise interactions, mixing speed*pH, natural acid concentration*mixing speed, and natural acid concentration*pH have effects of 10, 0, and 0%, respectively. The triple interaction, natural acid concentration*mixing speed*pH, has an effect of 0%. It is clear that the most influential factor in the crystallization process affecting purity is “pH”.

#### 3.2.2 Yield of AMCT Crystals

**Figure 8:**
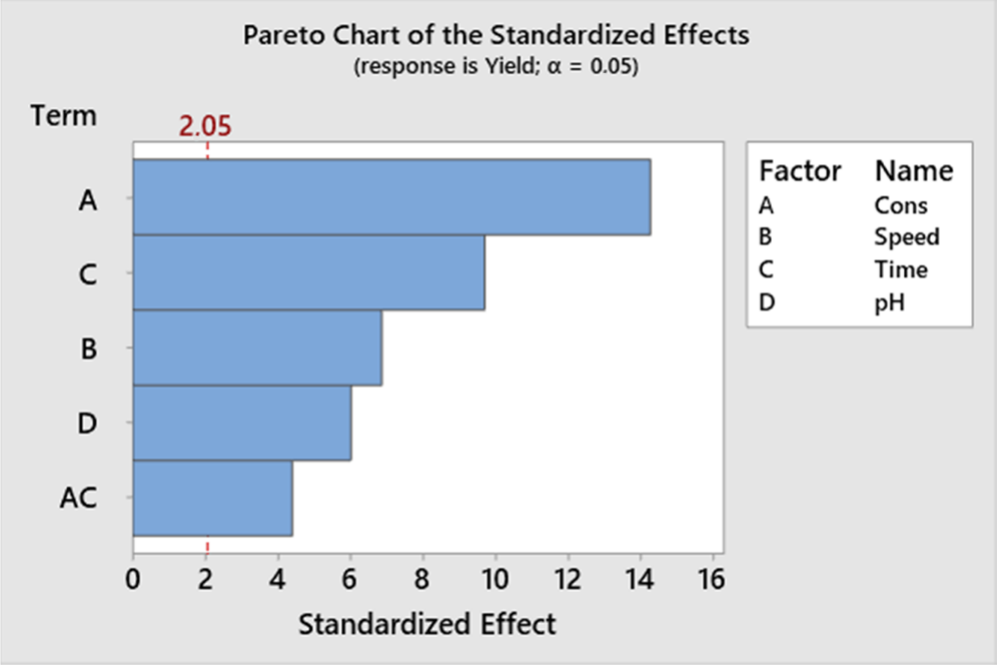
Pareto chart for the AMCT yield.

The total yield in the study conducted using natural acid can be expressed as the ratio of the amount of product obtained after washing to the total amount of material used. The obtained AMCT yield values were modeled by using a full factorial design model in Minitab software, and the analysis of variance (ANOVA) values are provided in Table 5. In this table, the P-value column considers a 95% confidence level. The results indicate that the factors “Concentration”, “Speed”, “Time”, and “pH”, as well as the interaction “Concentration*Time”, have a significant effect on yield. Additionally, when examining the Pareto chart shown in Figure 8, it can be observed that factors A–C cross the red dotted line, indicating their significance at a 95% confidence level. The summary of the model for the yield values is presented in Table 6. The standard deviation of residuals (S) is 2.52. The *R*^2^ value indicates how much of the variation in the response can be explained by the model and is 93.71%, which is considered good. The remaining 6.29% of the variation cannot be explained by the model. The adjusted *R*^2^ value (*R*^2^ (adj)) is 92.13%. 2 represents the regression equation in uncoded units.
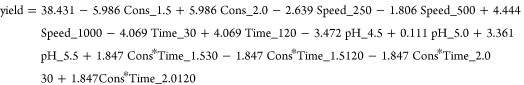
2

**Table 5:** Analysis of Variance for Yield

source	DF	adj SS	adj MS	*F*-value	*P*-value
model	7	2649.10	378.45	59.55	0.000
linear	6	2526.30	421.05	66.25	0.000
cons	1	1290.00	1290.01	202.99	0.000
speed	2	359.70	179.86	28.30	0.000
time	1	596.20	596.17	93.81	0.000
pH	2	280.40	140.19	22.06	0.000
2-way interactions	1	122.80	122.84	19.33	0.000
cons*time	1	122.80	122.84	19.33	0.000
error	28	177.90	6.36		
total	35	2827.10			

**Table 6:** Model Summary for Yield

*S*	*R*^2^	*R*^2^ (adj)	*R*^2^ (pred)
2.52	93.71%	92.13%	89.60%

**Figure 9:**
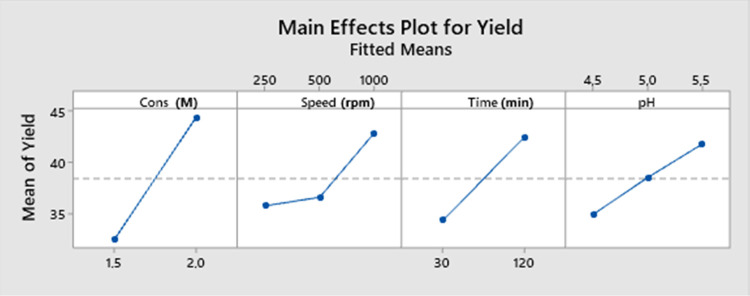
Main effect graph for AMCT yield.

The main effect graph for the AMCT yield (Figure 9) examines the independent effects of natural acid concentration and process variables such as mixing speed, crystallization time, and pH on the AMCT yield. The graph clearly shows the relationship between acid concentration and yield, indicating that as the acid concentration increases, the yield value also increases. Additionally, positive effects of crystallization time, pH value, and mixing speed on yield can be observed. Increasing these variables leads to an increase in yield.

**Figure 10:**
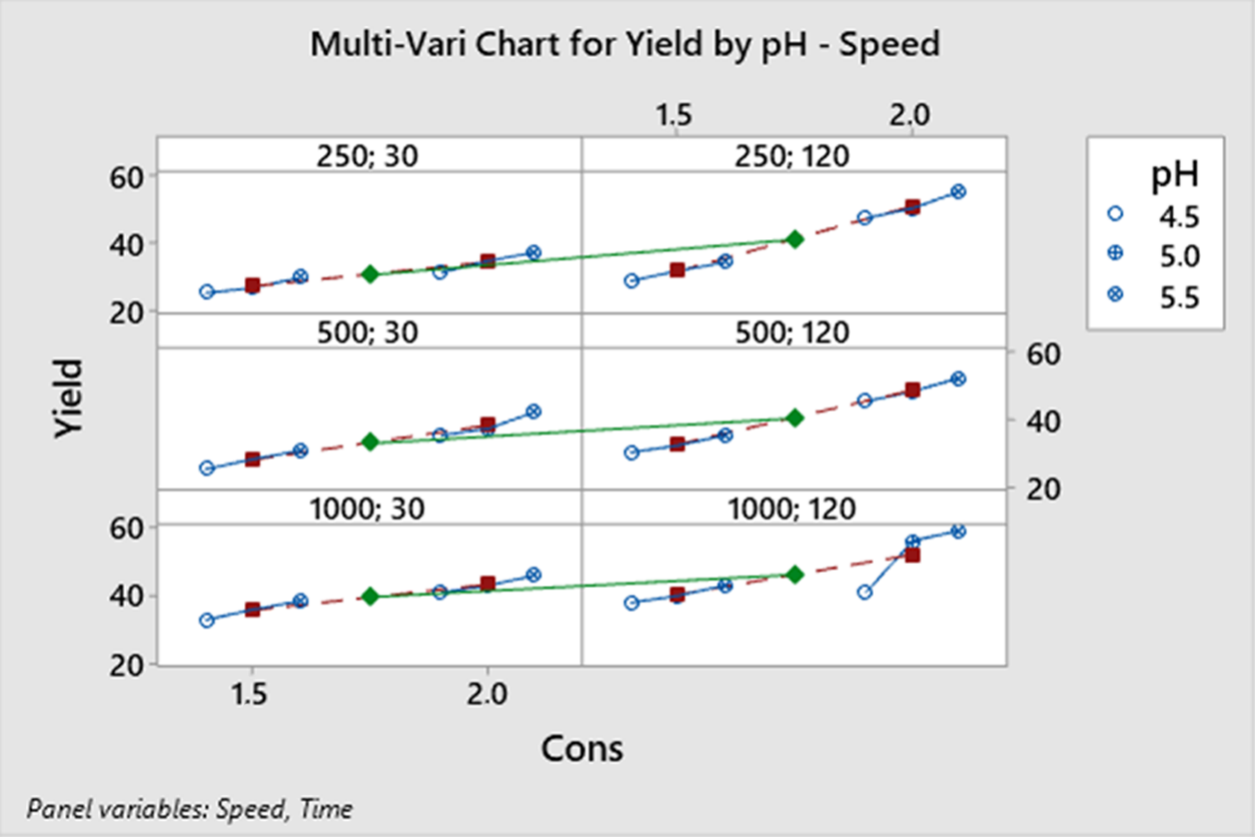
Multi-vari chart for the AMCT yield.

Figure 10 presents the relationships between all variables. According to Figure 10, the highest yield of 59% was achieved with a 2.0 M acid concentration, a pH of 5.5, a stirring speed of 1000 rpm, and a crystallization time of 120 min.

**Figure 11:**
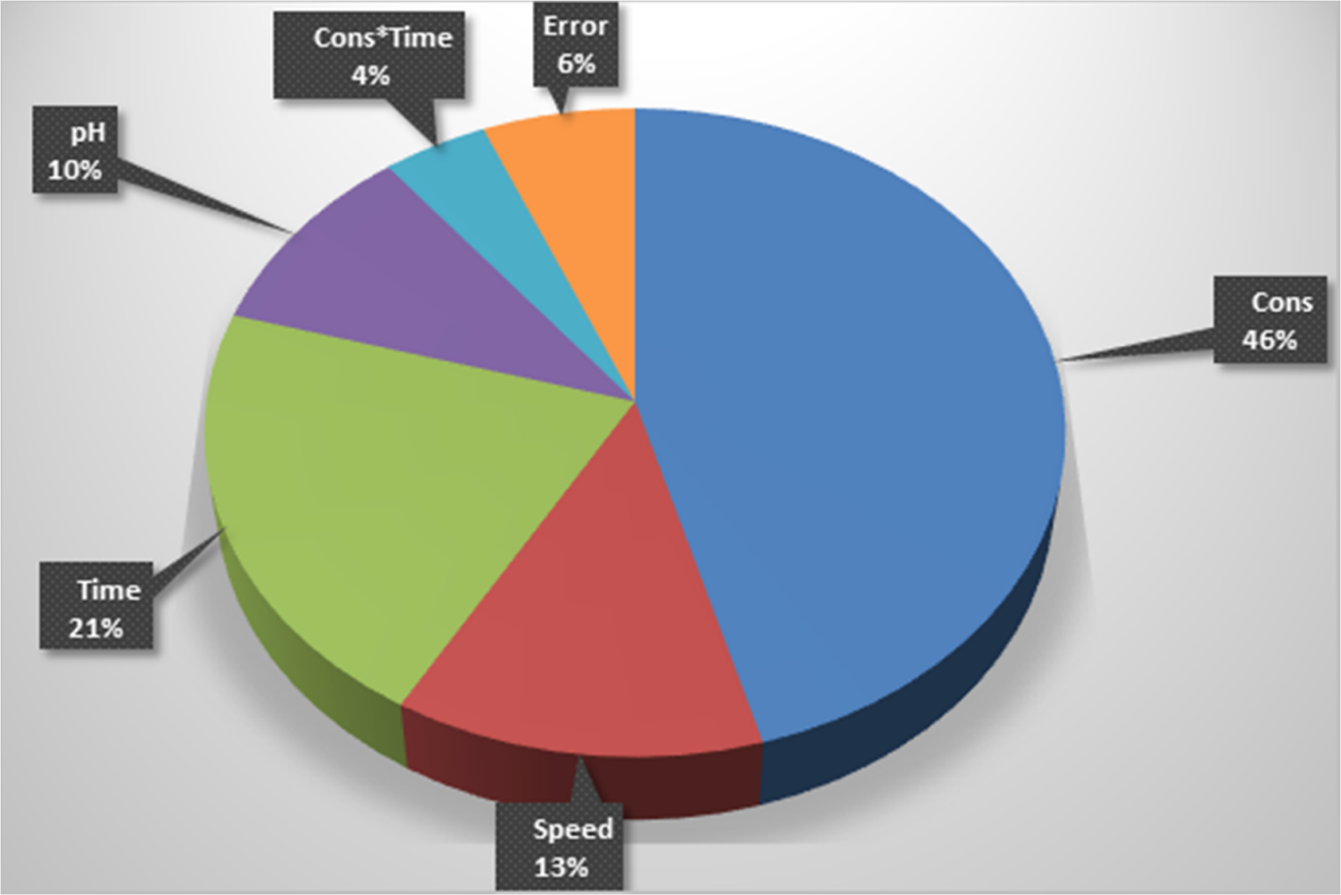
Factors affecting the AMCT yield.

The factors influencing AMCT yield are shown in the pie chart in Figure 11. pH has a 10% effect, stirring speed 13%, natural acid concentration 46%, and crystallization time 21% on the total variability. As for the pairwise interactions, Concentration*Time accounts for 4% of the effect, and the unexplained portion expressed as “error” accounts for 6%. It is clearly seen within the model framework that “Concentration” is the most influential factor affecting yield.

#### 3.2.3 Optimization of AMCT Purity and Yield Responses

**Figure 12:**
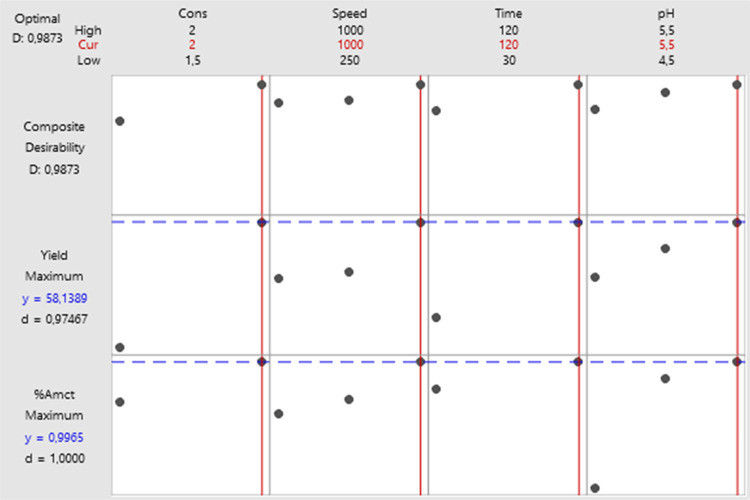
Response optimization for AMCT purity and yield.

To optimize the purity and total yield of AMCT crystallized with natural acid, input variables such as the natural acid concentration, mixing speed, crystallization time, and pH combination were used. The response optimizer module of the Minitab software package was employed in the study for optimization, and the most suitable solution was determined. As shown in the multiple response prediction (Table 7), a natural acid concentration of 2.0 M was preferred, while the process conditions were optimized to include a mixing speed of 1000 rpm, a crystallization time of 120 min, and a pH of 5.5. The obtained desirability value (D) was 0.98 (Figure 12).

**Table 7:** Multiple Response Prediction

variable	setting
cons	2
speed	1000
time	120
pH	5.5

## 4 Conclusions

This article presents a significant study that focuses on the removal of 4-HPG, an impurity added to the crystal lattice of amoxicillin trihydrate (AMCT), whose molecular structure is similar to that of AMCT. The impurity is removed from its crystalline structure using environmentally friendly citric acid. The study sheds light on the dissolution stage of AMCT, which is the initial step in both batch and continuous crystallization processes for AMCT. This study is the first to highlight the importance of citric acid utilization in the crystallization process from a comprehensive perspective. The effects of citric acid on the nucleation and growth of AMCT crystals were studied and analyzed. The resulting crystal structures exhibit dimensions that are highly desirable in the pharmaceutical industry. These dimensions were achieved without the need for additional purification steps.^32^ Moreover, the use of citric acid is expected to have positive contributions to various aspects of the AMCT crystallization process, including product design, purification, and synthesis.

The study was designed using a two-level citric acid concentration, a three-level mixing speed, a three-level pH, and a two-level crystallization time. Experiments were conducted by following a full factorial design. The purity and yield values of AMCT were analyzed using a multivariate table, and process parameters such as the citric acid concentration, pH, stirring speed, and crystallization time were examined. Instead of using the commonly preferred acids in the crystallization process, different concentrations of citric acid were employed. This consideration took into account the balance among cost, purity, yield, and environmental impact. The results were then compared. The highest purity value was achieved under the conditions of pH of 5.5, stirring speed of 1000 rpm, and crystallization time of 120 min, yield of 99.64%. Additionally, the highest yield of 59% was obtained with a 2.0 M acid concentration, pH of 5.5, stirring speed of 1000 rpm, and crystallization time of 120 min.

Furthermore, this study highlights the distinct nature of the crystallization process compared to other procedures documented in the literature and industry. The notable difference lies in the absence of any environmentally harmful chemicals employed during the process. As a result, this study exemplifies an exemplary green process, setting it apart within the existing body of the literature.
